# Bidirectional Longitudinal Associations Between Problematic Social Media Use and Emotional Symptoms in Adolescents: Results From a Random Intercept Cross‐Lagged Panel Model

**DOI:** 10.1002/jad.70237

**Published:** 2026-07-22

**Authors:** Chiara Remondi, Antonino La Tona, Laura Salerno, Rita Aiello, Valeria Donisi, Silvia Poli, Elide Francesca De Caro, Elisa Delvecchio, Agostino Brugnera

**Affiliations:** ^1^ Department of Psychology and Health Sciences Pegaso Telematic University Naples Italy; ^2^ Department of Human and Social Sciences University of Bergamo Bergamo Italy; ^3^ Department of Psychology, Educational Science and Human Movement University of Palermo Palermo Italy; ^4^ Department of Neuroscience, Biomedicine and Movement Science University of Verona Verona Italy; ^5^ Department of Philosophy, Social Sciences and Education University of Perugia Perugia Italy

**Keywords:** adolescence, emotional symptoms, longitudinal study, problematic social media use, random intercept cross‐lagged panel model

## Abstract

**Introduction:**

Problematic social media use (PSMU) and emotional symptoms are consistently associated during adolescence; however, the directionality of this relationship remains debated.

**Methods:**

The present study examined the bidirectional longitudinal associations between PSMU and emotional symptoms in a large sample of Italian adolescents using a Random Intercept Cross‐Lagged Panel Model (RI‐CLPM), which disentangles stable between‐person differences from within‐person changes over time. A total of 1562 adolescents (baseline M_age_ = 16.36 years, 56.7% female) participated in a three‐wave longitudinal study across 1 year and completed self‐report measures of emotional symptoms and PSMU at each wave.

**Results:**

At the between‐person level, adolescents who consistently reported higher levels of PSMU also reported persistently higher levels of emotional symptoms, indicating a stable dispositional association between the two constructs. At the within‐person level, higher‐than‐usual levels of PSMU were concurrently associated with higher‐than‐usual levels of emotional symptoms. Most notably, significant bidirectional spill‐over effects emerged: within‐person increases in PSMU predicted subsequent increases in emotional symptoms, and increases in emotional symptoms predicted subsequent increases in PSMU. Emotional symptoms also demonstrated significant carry‐over effects across waves.

**Conclusions:**

These findings highlight the importance of disaggregating within‐person and between‐person effects and support the conceptualization of the association between PSMU and emotional symptoms as a dynamic feedback loop during adolescence.

1

Social media use has become ubiquitous among adolescents, permeating multiple domains of young people's daily lives worldwide. As digital technologies continue to evolve, screen‐based devices increasingly afford a wide range of activities, with social networking sites emerging as one of the most popular forms of engagement among young people. Indeed, platforms such as Instagram, TikTok, and Snapchat are now deeply embedded in adolescents' everyday routines (Keles et al. 2020).

Alongside this widespread use, growing concerns have been raised regarding the association between social media use and adolescents' well‐being. In fact, recent evidence highlights that among teens who report being at least somewhat concerned about the well‐being of young people today, 22% identify social media as the primary contributing factor (Pew Research Center 2025). These concerns are particularly pronounced when social media use becomes excessive or dysregulated.

Problematic social media use (PSMU) refers to a pattern of persistent and compulsive engagement with social media platforms that resembles behavioral addiction and is marked by diminished control over use (van den Eijnden et al. 2016). Empirical evidence has linked PSMU to a range of adverse psychological and behavioral outcomes, including emotional symptoms (e.g., depression and anxiety), sleep disturbances, academic difficulties, and reduced quality of life (see Pazdur et al. 2025 for a review). In particular, adolescents with PSMU consistently report higher levels of depressive and anxious symptoms, with accumulating evidence suggesting that this association may be bidirectional (Lopes et al. 2022).

From one perspective, both theoretical models and empirical evidence suggest that emotional symptoms may precede and contribute to the greater severity of PSMU. According to the Cognitive–Behavioral Model (Davis 2001) and the Compensatory Internet Use Theory (CIUT; Kardefelt‐Winther 2014), individuals experiencing psychological distress may engage excessively with digital media as a maladaptive coping strategy to regulate negative emotions and escape from real‐life problems. In this view, psychosocial maladjustment fosters maladaptive cognitions—such as the belief that Internet use can effectively alleviate one's difficulties—thereby reinforcing excessive online engagement. This perspective has been supported by a substantial body of cross‐sectional research, as well as longitudinal studies examining temporal precedence (e.g., Raudsepp and Kais 2019; Zhou et al. 2023). For instance, in a three‐wave longitudinal study of Chinese undergraduate students, Zhou et al. (2023) found that higher levels of depression and anxiety at earlier time points significantly predicted greater PSMU severity at later assessments, whereas earlier PSMU did not predict subsequent depressive or anxiety symptoms. Similarly, in a two‐wave longitudinal study among Spanish adolescents, Gámez‐Guadix (2014) showed that prior psychological distress constituted a risk factor for the development of problematic Internet use 1 year later.

In contrast, other theoretical frameworks and empirical findings suggest the opposite direction of influence, whereby PSMU may be a key precursor of emotional symptoms (Beard 2005; Kraut et al. 1998). In particular, the Displacement Hypothesis (Kraut et al. 1998) posits that excessive smartphone or Internet use displaces protective activities—such as face‐to‐face social interactions, physical activity, and adequate sleep—that are crucial buffers against emotional distress. By occupying time that would otherwise be spent engaging with family and friends, excessive Internet use may undermine social development and increase the risk of social isolation, depression, and loneliness. Supporting this perspective, Lapierre et al. (2019), in a two‐wave longitudinal study of US adolescents aged 17−20 years, found that smartphone dependency predicted increased depressive symptoms 3 months later, whereas depressive symptoms at baseline did not predict subsequent smartphone dependency. Taken together, individuals with PSMU may develop mental health problems because of their drained coping resources, compromised social functioning, and decreased self‐control.

To date, although it is accepted that the association between PSMU and emotional symptoms may be described as a dynamic *feedback loop* (e.g., Slater 2015), whereby depressive or anxiety symptoms guide selective exposure to symptom‐relevant content that may exacerbate emotional distress over time, only a limited number of studies have empirically tested this process using appropriate longitudinal models (e.g., Di Blasi et al. 2022; Chang et al. 2022).

To our knowledge, only a few studies have implemented the random intercept cross‐lagged panel model (RI‐CLPM; Hamaker et al. 2015), and findings from these studies have been mixed regarding the directional associations between PSMU and emotional symptoms. For instance, Chang et al. (2022), in a study examining the bidirectional associations between PSMU and psychological distress among university students, found that the association between PSMU and anxiety was reciprocal, such that adolescents with higher levels of PSMU would exhibit higher levels of anxiety and, in turn, higher levels of anxiety would predict higher levels of PSMU over time. However, while higher levels of PSMU predicted increases in depressive symptoms, depressive symptoms did not predict subsequent PSMU. Similarly, in a study examining the bidirectional relationships between PSMU and social anxiety among adolescents, Zhang et al. (2025) demonstrated the mutual predictability between social anxiety and PSMU, such that previous PSMU would heighten subsequent social anxiety, and vice versa. Conversely, in a longitudinal study among adult participants, Di Blasi et al. (2022) found that although adults with higher PSMU consistently exhibited higher levels of psychological distress, no significant bidirectional associations emerged between psychological distress and subsequent changes in PSMU over time. The inconsistent pattern of within‐person effects underscores the need for further longitudinal research in adolescent populations using models capable of disentangling stable between‐person from dynamic within‐person differences.

Therefore, the present study aims to explore the bidirectional longitudinal associations between PSMU and emotional symptoms by implementing the RI‐CLPM to answer three research questions: (1) Are stable (i.e., trait‐like) parts of adolescents' PSMU and emotional symptoms related to each other? (2) Are higher‐than‐expected levels of PSMU associated with higher‐than‐expected levels of emotional symptoms at a given time point? and (3) Are higher‐than‐expected levels of PSMU at a given time point associated with subsequent higher‐than‐expected levels of emotional symptoms, and vice versa?

First, at the between‐person level, we hypothesize that stable individual differences in PSMU will be positively associated with stable individual differences in emotional symptoms. Specifically, adolescents who consistently report higher levels of PSMU across time are expected to also report higher levels of psychological distress, and vice versa.

Second, given the mixed evidence regarding longitudinal within‐person associations between PSMU and emotional symptoms, we explore whether within‐person increases in emotional symptoms predict subsequent within‐person increases in PSMU, and whether within‐person increases in PSMU predict subsequent within‐person increases in emotional symptoms.

## 2 Materials and Methods

### 2.1 Participants

A total of 1562 Italian adolescents participated in the study. Age and sex distributions at each wave are reported in Table 1. Students were recruited from upper secondary schools located across five Italian regions (Lombardy, Veneto, Umbria, Marche, and Sicily).

**Table 1:** Age and sex distribution across the three study waves in our sample of adolescents.

	Age		Sex (female)	
Time point	*N*	Mean (SD)	*N* (total)	Valid %
T0	1439	16.364 (1.158)	855 (1507)	56.7%
T1	1196	16.452 (1.242)	677 (1198)	56.5%
T2	1090	17.517 (1.226)	620 (1103)	56.2%

Abbreviation: SD = standard deviation.

Most participants were born in Italy (93.7%, *n* = 1282). Most adolescents also reported that their parents were born in Italy (77.9% of mothers and 81.1% of fathers), indicating that the sample predominantly comprised youth without a migration background.

Regarding social media habits at baseline, TikTok emerged as the platform most frequently used by participants (49.6%, *n* = 678), followed by Instagram (41.3%, *n* = 564), other platforms (e.g., Twitch; 6.4%, *n* = 87), Snapchat (0.4%, *n* = 6), Facebook (0.4%, *n* = 6), and Twitter (0.4%, *n* = 5). Finally, 20 participants self‐reported a very limited use of social media (1.5%).

The study was conducted in accordance with ethical standards and was approved by the regional ethics committee of Umbria, Italy (Prot. *N*. 27844/23/AV, November 28, 2023). Written informed consent was obtained from both parents and/or legal guardians prior to adolescents' participation; participants aged 18 provided their own consent.

### 2.2 Measures

#### 2.2.1 Emotional Symptoms

The Italian version of the Strengths and Difficulties Questionnaire (SDQ; Essau et al. 2012; Goodman 1997) is a 25‐item self‐report of strengths and difficulties in adolescents' psychological adjustment. The present study focused on the Emotional Symptoms subscale (5 items), which measures anxiety, depressive symptoms, fears, and somatic complaints (e.g., *I am often unhappy, depressed, or tearful*). Each item uses a 3‐point Likert‐type scale (from 0 = *not true*, to 2 = *certainly true*), with higher summed scores indicating more severe emotional symptoms. In the current study, the internal consistency of the scale was good at all time points (T0 *ω* = 0.75; T1 *ω* = 0.77; T2 *ω* = 0.77).

#### 2.2.2 PSMU

The Italian version of the Bergen Social Media Addiction Scale (BSMAS; Monacis et al. 2017; Andreassen et al. 2016) is a 6‐item self‐report measure assessing six core components of addiction (i.e., salience, mood modification, tolerance, withdrawal, conflict, and relapse) related to social media use during the past year (e.g., *You use social media in order to forget about personal problems*). Items are rated on a 5‐point Likert‐type scale (from 1 = *very rarely*, to 5 = *very often*), with higher summed scores reflecting greater PSMU. In the current study, internal consistency was good across all time points (T0 *ω* = 0.75; T1 *ω* = 0.76; T2 *ω* = 0.78).

### 2.3 Procedure

Data were collected from December 2023 to February 2025 as part of a larger longitudinal study investigating risk and protective factors of PSMU among Italian mid‐adolescents (Donisi et al. 2024). Participants completed an online survey during school hours at three time points, with the following median administration dates: T0 (December 2023), T1 (June 2024), and T2 (December 2024).

Researchers administered the online survey in classroom settings. Inclusion criteria were: (i) age between 14 and 18 years, and (ii) adequate comprehension of spoken and written Italian. Participants did not receive any compensation or incentives for taking part in the study.

### 2.4 Statistical Analysis

We first tested the data for univariate normality and outliers and examined descriptive statistics and zero‐order correlations among all study variables.

Bidirectional associations between PSMU and emotional symptoms were examined using a RI‐CLPM based on observed scale scores. Models were estimated using Maximum Likelihood with standard errors robust to non‐normality, with missing data handled through full information maximum likelihood (FIML). Good model fit was evaluated using the following criteria: a root mean square error of approximation (RMSEA) ≤ 0.06; a comparative fit index (CFI) and a Tucker‐Lewis index (TLI) ≥ 0.95, and a standardized root mean square residual (SRMR) ≤ 0.05 (Hu and Bentler 1999).

Although the model was based on observed variables, the RI‐CLPM specifies latent random intercepts and latent within‐person components, thereby disentangling the between‐person (trait‐like) and within‐person (state‐like) associations between emotional symptoms and PSMU (Hamaker et al. 2015). At the between‐person level, the association between PSMU and emotional symptoms represents the dispositional association between the two variables (e.g., do adolescents with higher PSMU, in general, report higher emotional symptoms?). At the within‐person level, the effects refer to the relations between PSMU and emotional symptoms in terms of deviations from the person's mean score (Hamaker et al. 2015). Specifically, the within‐person effects capture three types of relations: (a) simultaneous, time‐specific associations, which reflect whether higher‐than‐usual levels of PSMU are related to higher‐than‐usual levels of emotional symptoms at the same time point; (b) carry‐over effects, which reflect whether higher‐than‐usual levels of a construct at one time point predict higher‐than‐usual levels of the same construct at the subsequent time point; and (c) spill‐over effects, which reflect whether higher‐than‐usual levels of one construct at one time point predict higher‐than‐usual levels of the other construct at the subsequent time point.

Two RI‐CLPMs were estimated: one allowing autoregressive and cross‐lagged paths to vary freely over time, and one constraining these paths to equality across time points. Following Hamaker et al. (2015), the constrained model was preferred over the unconstrained model when imposing equality constraints did not significantly worsen model fit, as it provided a more parsimonious representation of the longitudinal associations. Nested models were compared using Satorra−Bentler scaled *χ* ^2^ difference tests. At each time point, both emotional symptoms and PSMU were regressed on biological sex to control for its confounding effects.

All analyses were conducted using Mplus 8.11 (Muthén and Muthén 2024) and SPSS 29.0.1 (IBM Corp 2023). Effect sizes, including those for cross‐lagged effects, were interpreted according to guidelines (Orth et al. 2024). All tests were two‐sided, and a *p* < 0.05 was deemed significant.

## 3 Results

Descriptive statistics and zero‐order correlations are presented in Tables 2 and 3, respectively. Adolescents who used social media in a more problematic way reported greater emotional symptoms across the waves; further, female adolescents reported higher levels of both PSMU and emotional symptoms than males.

**Table 2:** Descriptives for emotional symptoms and PSMU, separately for each time point, in our sample of adolescents.

	T0				T1				T2			
Variable	*N*	M (SD)	Sk	Kr	*N*	M (SD)	Sk	Kr	*N*	M (SD)	Sk	Kr
Emot. Symp.	1362	9.160 (2.652)	0.28	−0.81	1141	9.249 (2.689)	0.27	−0.84	1099	9.243 (2.664)	0.21	−0.87
PSMU	1292	14.979 (4.790)	0.39	−0.25	1089	15.105 (4.754)	0.31	−0.16	1060	14.644 (4.847)	0.20	−0.42

Abbreviations: Emot. Symp. = emotional symptoms, Kr = kurtosis, M (SD) = mean (standard deviation), PSMU = problematic social media use, Sk = skewness.

**Table 3:** Zero‐order correlations among all variables of interest.

Variable	1.^a^	2.	3.	4.	5.	6.
1. Sex (female)^a^	\					
2. Emot. Symp. T0	0.464**	\				
3. Emot. Symp. T1	0.427**	0.758**	\			
4. Emot. Symp. T2	0.411**	0.691**	0.746**	\		
5. PSMU T0	0.252**	0.378**	0.371**	0.351**	\	
6. PSMU T1	0.243**	0.320**	0.396**	0.324**	0.638**	\
7. PSMU T2	0.219**	0.270**	0.367**	0.426**	0.586**	0.619**

*Note:* ^a^Point‐biserial correlation coefficients; **p < 0.001.

Abbreviations: Emot. Symp. = emotional symptoms, PSMU = problematic social media use.

As shown in Table 4, both the unconstrained model, with autoregressive and cross‐lagged paths allowed to vary freely over time, and the constrained model, with these paths constrained to equality across time, demonstrated good fit to the data. Specifically, the constrained model showed excellent fit (CFI = 0.997, TLI = 0.993, RMSEA = 0.026, SRMR = 0.020). The Satorra−Bentler scaled *χ* ^2^ difference test indicated that constraining the paths to equality across time did not worsen model fit, Δ*χ* ^2^(8) = 12.83, *p* = 0.118. Accordingly, the constrained model was retained as the final model; detailed parameter estimates, including standard errors and 95% confidence intervals, are reported in Table 5, whereas Figure 1 provides a graphical summary of the main findings.

**Table 4:** Fit indices for the unconstrained and the constrained Random Intercept Cross‐Lagged Panel Model (RI‐CLPM).

Model	*χ* ^2^	df	scf	*p*	AIC	BIC	SSABIC	CFI	TLI	RMSEA [90% CIs]	SRMR
Unconstrained	6.036	1	0.8571	0.014	33,796.25	33,966.33	33,864.68	0.998	0.963	0.058 [0.021, 0.106]	0.009
Constrained	18.203	9	1.0074	0.033	33,793.41	33,920.98	33,844.74	0.997	0.993	0.026 [0.007, 0.043]	0.020

Abbreviations: *χ* ^2^ = chi‐square statistic, AIC = Akaike information criterion, BIC = Bayesian information criterion, CFI = comparative fit index, df = degrees of freedom, *p* = *p*‐value, RMSEA [90% CIs] = root mean square error of approximation [90% confidence intervals], scf = scaling correction factor, SRMR = standardized root mean square residual, SSABIC = sample‐size adjusted Bayesian information criterion, TLI = Tucker‐Lewis index.

**Table 5:** Standardized parameter estimates for the Random Intercept Cross‐Lagged Panel Model of problematic social media use and emotional symptoms in adolescents.

Parameter	Std. Estimate	SE	95% CI	*p* value
Between‐level correlation				
PSMU ↔ Emot. Symp.	0.359	0.052	[0.257, 0.460]	< 0.001
Within‐level				
*Correlations*				
T0 PSMU ↔ T0 Emot. Symp.	0.240	0.070	[0.103, 0.377]	0.001
T1 PSMU ↔ T1 Emot. Symp.	0.266	0.061	[0.147, 0.384]	< 0.001
T2 PSMU ↔ T2 Emot. Symp.	0.316	0.043	[0.231, 0.401]	< 0.001
*Carry‐over effects:* PSMU → PSMU				
T0 PSMU → T1 PSMU	0.153	0.080	[−0.004, 0.309]	0.056
T1 PSMU → T2 PSMU	0.145	0.080	[−0.011, 0.302]	0.068
*Carry‐over effects:* Emot. Symp. → Emot. Symp.				
T0 Emot. Symp. → T1 Emot. Symp.	0.236	0.059	[0.120, 0.351]	< 0.001
T1 Emot. Symp. → T2 Emot. Symp.	0.249	0.067	[0.117, 0.380]	< 0.001
*Spill‐over effects:* PSMU → Emot. Symp.				
T0 PSMU → T1 Emot. Symp.	0.162	0.058	[0.050, 0.275]	0.005
T1 PSMU → T2 Emot. Symp.	0.161	0.056	[0.051, 0.270]	0.004
*Spill‐over effects:* Emot. Symp. → PSMU				
T0 Emot. Symp. → T1 PSMU	0.184	0.057	[0.073, 0.295]	0.001
T1 Emot. Symp. → T2 PSMU	0.187	0.060	[0.070, 0.304]	0.002

Abbreviations: CI = confidence interval, Emot. Symp. = emotional symptoms, PSMU = problematic social media use, SE = standard error, Std. Estimate = standardized estimate (*r* for correlations; *β* for regression paths).

**Figure 1:**
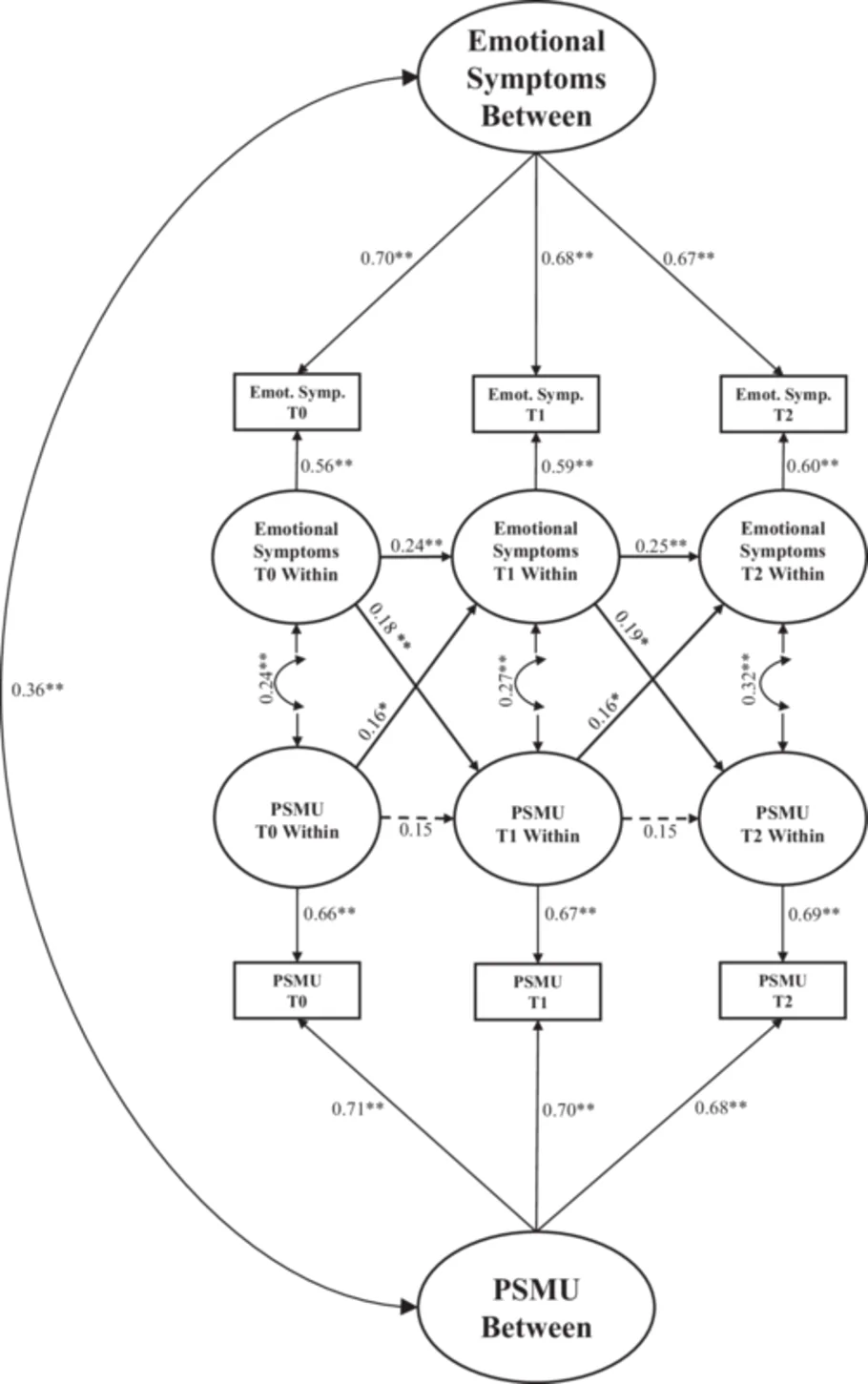
Standardized parameter estimates from the constrained Random Intercept Cross‐Lagged Panel Model of problematic social media use and emotional symptoms in adolescents. Coefficients include between‐person correlation, within‐time correlations, autoregressive and cross‐lagged paths, and factor loadings for the latent between‐person and within‐person components. Emot. Symp. = Emotional symptoms, PSMU = Problematic social media use. *p < 0.05, **p < 0.001.

At the between‐person level, a significant cross‐time association was found between PSMU and emotional symptoms, suggesting that adolescents who consistently reported higher levels of PSMU also tended to report persistently higher levels of emotional symptoms (*r* = 0.36, *p* < 0.001).

At the within‐person level, PSMU was significantly associated with emotional symptoms at each time point (T0, *r* = 0.24, *p* = 0.001; T1, *r* = 0.27, *p* < 0.001; and T2, *r* = 0.32, *p* < 0.001). These findings suggest that adolescents reporting higher‐than‐usual levels of PSMU at a given time point also tended to report higher‐than‐usual emotional symptoms at the same time point.

Carry‐over effects were significant only for emotional symptoms, indicating that adolescents with higher‐than‐expected levels of emotional symptoms at a given time point were also more likely to report elevated emotional symptoms at the subsequent time point, reflecting a greater persistence of emotional distress over time (T0 to T1, *β* = 0.24, *p* < 0.001; T1 to T2, *β* = 0.25, *p* < 0.001). In contrast, carry‐over effects for PSMU were not statistically significant, suggesting that fluctuations in PSMU may be more transient and less enduring than emotional symptoms (T0 to T1, *β* = 0.15, *p* = 0.056; T1 to T2, *β* = 0.15, *p* = 0.068).

Finally, significant bidirectional spill‐over effects emerged between PSMU and emotional symptoms. Greater PSMU at T0 predicted greater emotional symptoms at T1 (*β* = 0.16, *p* = 0.005), and greater PSMU at T1 predicted greater emotional symptoms at T2 (*β* = 0.16, *p* = 0.004). In turn, greater emotional symptoms at T0 predicted subsequent increases in PSMU at T1 (*β* = 0.18, *p* = 0.001), and greater emotional symptoms at T1 predicted greater PSMU at T2 (*β* = 0.19, *p* = 0.002). These effects were large in magnitude (Orth et al. 2024) and suggest the presence of a reciprocal process whereby increases in PSMU and emotional symptoms mutually reinforce one another over time.

## 4 Discussion

Given prior considerations regarding the bidirectional relations between PSMU and emotional symptoms (Slater 2015), the present study extended previous work by providing a longitudinal analysis of the between‐ and within‐person associations of PSMU and emotional symptoms in a large sample of Italian adolescents using a RI‐CLPM. Overall, our results indicated a bidirectional association between PSMU and emotional symptoms over time, suggesting that increases in adolescents' PSMU predicted increases in emotional symptoms, and, reciprocally, increases in emotional symptoms predicted increases in PSMU over time.

First, in accordance with Hypothesis 1, we found a significant positive association between PSMU and emotional symptoms at the between‐person level, indicating that adolescents who consistently report higher levels of PSMU across time also tend to report higher levels of emotional distress. Our results on the trait‐like association between PSMU and emotional symptoms are in line with prior longitudinal (Di Blasi et al. 2022) and meta‐analytic findings (Huang 2022), suggesting that individuals experiencing persistent psychological distress may be more prone to engage in excessive online behaviors as a maladaptive coping strategy. From this perspective, PSMU may represent a relatively stable behavioral expression of broader psychosocial maladjustment, whereas emotional symptoms reflect enduring affective vulnerabilities that co‐occur with, and potentially contribute to, the maintenance of dysregulated social media use. In fact, although engagement with social media may initially serve adaptive functions for adolescents, such as fostering social connection, repeated reliance on these platforms for regulating affect and emotions may, over time, undermine the use of adaptive coping strategies. As adolescents increasingly turn to social media to manage negative emotions, this pattern of use may become reinforced, thereby increasing the risk of developing problematic or compulsive engagement (Gámez‐Guadix 2014; Kim et al. 2009). Importantly, the between‐person association should not be interpreted as evidence of causal directionality at the trait level, but rather as an indication that stable emotional symptoms and stable problematic social media engagement tend to co‐occur as part of a pattern of psychosocial maladjustment.

Most notably, at the within‐person level, consistent with Hypothesis 2, the present study found evidence of bidirectional spill‐over effects between PSMU and emotional symptoms. Higher‐than‐usual levels of PSMU at one point predicted subsequent increases in emotional symptoms, and, reciprocally, higher‐than‐usual levels of emotional symptoms predicted subsequent increases in PSMU. These reciprocal effects were observed consistently across waves and suggest that fluctuations in problematic social media engagement are meaningfully linked to fluctuations in emotional functioning beyond stable predispositions. This reciprocal pattern provides empirical support for conceptualizing the association between PSMU and emotional symptoms as a dynamic feedback loop, consistent with bidirectional theoretical frameworks, such as the Reinforcing Spirals Model (Slater 2015), which proposes that depressed or anxious individuals would selectively seek content that may reinforce their symptoms, and that depressive or anxiety‐inducing content could in turn further intensify emotional symptoms. In other words, our findings suggest the presence of a bidirectional process whereby adolescents experiencing elevated emotional symptoms may turn to online environments to regulate negative affect, alleviate feelings of anxiety, or temporarily escape distressing thoughts and emotions (Nesi et al. 2018). At the same time, excessive or problematic engagement with social media may further exacerbate emotional difficulties, as social media environments can also represent a source of stress through negative social interactions, social comparison, and communication overload (Vannucci et al. 2017).

Interestingly, while PSMU did not show significant temporal stability at the within‐person level, emotional symptoms displayed significant carry‐over effects. In other words, adolescents with higher‐than‐expected levels of emotional symptoms at a given time point also tended to report higher‐than‐expected levels of emotional symptoms at the subsequent time point. A possible explanation for the observed pattern of findings may lie in the developmental characteristics of adolescence. This period is marked by heightened emotion dysregulation (Beauchaine and Hinshaw 2017) and increased negative emotionality (Sallquist et al. 2009), reflecting the profound biological, cognitive, social, and emotional transformations that occur during this stage (Lerner and Steinberg 2009; Silk et al. 2003). As regulatory capacities are still consolidating, adolescents may experience greater emotional reactivity and reduced ability to effectively modulate distress. Within this context, the significant carry‐over effects observed for emotional symptoms suggest that deviations in distress tend to persist over time, rather than dissipate quickly. In contrast, the non‐significant carry‐over effects of PSMU suggest that increases in PSMU may be less likely to persist across measurement occasions. One possible explanation is that adolescents' problematic engagement with social media may be more strongly influenced by transient contextual and situational factors, such as peer dynamics, shifts in emotional well‐being, or changing patterns of online activities (Pouwels et al. 2025). As a result, deviations in PSMU may be more episodic over time, rather than reflecting a stable pattern that consistently carries over from one assessment to the next.

### 4.1 Strengths, Limitations, and Future Directions

The current study contributes to the field by using a large community sample of adolescents and an RI‐CLPM to disentangle stable between‐person differences from within‐person dynamics in the association between PSMU and emotional symptoms. Nevertheless, several limitations should be considered when interpreting these findings. First, all constructs were assessed through adolescents' self‐reports, which may have inflated associations due to shared method variance. Although reliability was adequate, future studies should combine self‐report measures with other forms of assessment, such as parent or peer reports. Second, the present study did not account for other personal and contextual factors that may shape the longitudinal association between emotional symptoms and PSMU. As highlighted in recent systematic reviews (e.g., Poli et al. 2025), PSMU is embedded within a broader constellation of intrapersonal (e.g., attachment, self‐control, personality traits) and interpersonal (e.g., peer and family relationships) susceptibility factors. Future studies should therefore include these variables to better capture the complexity of the developmental processes underlying the association between emotional symptoms and PSMU. Third, emotional symptoms were assessed using a multi‐item scale encompassing heterogeneous dimensions, including anxiety, depressive symptoms, fears, and somatic complaints. While this approach captures general emotional distress, it does not allow for the examination of potentially distinct longitudinal pathways for specific symptom domains. Future studies should therefore examine specific symptom domains separately (e.g., anxiety, depressive symptoms, somatic complaints), as each may show distinct longitudinal associations with PSMU in terms of strength, directionality, or underlying mechanisms. Finally, although the longitudinal design spanned 1 year, adolescence is a prolonged developmental period characterized by substantial changes in autonomy, peer orientation, and risk‐taking behaviors. Future research should extend the application of RI‐CLPM models across broader developmental transitions, including the shift from mid‐ to late adolescence, to determine whether reciprocal dynamics between emotional symptoms and problematic digital behaviors strengthen, weaken, or change in nature over time.

Despite these limitations, our findings provide useful practical insights. Specifically, our findings suggest that interventions aimed at reducing PSMU during adolescence should not only focus on PSMU but also promote adolescents' emotional well‐being. In line with this perspective, school‐based interventions targeting adolescents' emotional self‐regulatory abilities have shown promising results in reducing problematic smartphone and social network use (Favini et al. 2024). Moreover, parents, educators, clinicians, and policymakers should consider adolescents' emotional well‐being as a key target for the prevention of PSMU.

## 5 Conclusion

Overall, the present study examined the longitudinal interplay between PSMU and emotional symptoms during adolescence using a RI‐CLPM. The findings showed that these constructs are associated at both the between‐person and within‐person levels, with reciprocal within‐person effects over time. These findings suggest that PSMU and emotional symptoms operate within a dynamic feedback process during adolescence, whereby increases in one domain contribute to subsequent increases in the other.

From a theoretical perspective, the findings indicate that the association between these constructs unfolds through both stable and dynamic processes. At the between‐person level, adolescents who consistently exhibit higher levels of PSMU also display persistently elevated emotional distress, suggesting a stable co‐occurrence of behavioral and affective vulnerability. Moreover, at the within‐person level, fluctuations in one domain prospectively predict changes in the other, supporting the existence of a reciprocal feedback process between PSMU and emotional symptoms.

From a practical point of view, our findings demonstrate that intervention programs may benefit from integrated approaches that promote adolescents' emotional well‐being alongside healthier patterns of social media engagement.

## Author Contributions

**Chiara Remondi:** methodology, formal analysis, writing – original draft, writing – review and editing, data curation, conceptualization. **Antonino La Tona:** writing – original draft, writing – review and editing, data curation, investigation. **Laura Salerno:** conceptualization, data curation, project administration, writing – review and editing, writing – original draft, funding acquisition, supervision, investigation. **Rita Aiello:** data curation, writing – review and editing, investigation. **Valeria Donisi:** data curation, project administration, writing–original draft, writing – review and editing, funding acquisition, supervision, investigation, conceptualization. **Silvia Poli:** data curation, writing – review and editing, investigation. **Elide Francesca De Caro:** data curation, writing – review and editing, investigation. **Elisa Delvecchio:** data curation, project administration, writing – original draft, writing – review and editing, funding acquisition, supervision, investigation, conceptualization. **Agostino Brugnera:** data curation, project administration, writing – original draft, writing – review and editing, funding acquisition, supervision, formal analysis, methodology, investigation, conceptualization.

## Ethics Statement

The study was approved by the Regional Ethics Committee of Umbria (Comitato Etico Regionale Umbria, CER; Protocol No. 27844/23/AV, approved on November 8, 2023). The study was conducted in accordance with the Declaration of Helsinki.

## Consent

Written informed consent was obtained from both parents and/or legal guardians prior to adolescents' participation; participants aged 18 provided their own consent.

## Conflicts of Interest

The authors declare no conflicts of interest.

## Data Availability

The data that support the findings of this study are available on request from the corresponding author. The data are not publicly available due to privacy or ethical restrictions.

## References

[jad70237-bib-0001] Andreassen, C. S. , J. Billieux , M. D. Griffiths , et al. 2016. “The Relationship Between Addictive Use of Social Media and Video Games and Symptoms of Psychiatric Disorders: A Large‐Scale Cross‐Sectional Study.” Psychology of Addictive Behaviors 30, no. 2: 252–262. 10.1037/adb0000160. 26999354

[jad70237-bib-0002] Beard, K. W. 2005. “Internet Addiction: A Review of Current Assessment Techniques and Potential Assessment Questions.” Cyberpsychology & Behavior: The Impact of the Internet, Multimedia and Virtual Reality on Behavior and Society 8, no. 1: 7–14. 10.1089/cpb.2005.8.7. 15738688

[jad70237-bib-0003] Beauchaine, T. P. , and S. P. Hinshaw . 2017. Child and Adolescent Psychopathology. 3rd ed. John Wiley & Sons. 10.1002/9781394258932.

[jad70237-bib-0004] Chang, C. W. , R. Y. Huang , C. Strong , et al. 2022. “Reciprocal Relationships Between Problematic Social Media Use, Problematic Gaming, and Psychological Distress Among University Students: A 9‐Month Longitudinal Study.” Frontiers in Public Health 10: 858482. 10.3389/fpubh.2022.858482. PMC9024175 35462841

[jad70237-bib-0005] Davis, R. A. 2001. “A Cognitive–Behavioral Model of Pathological Internet Use.” Computers in Human Behavior 17, no. 2: 187–195. 10.1016/S0747-5632(00)00041-8.

[jad70237-bib-0006] Di Blasi, M. , L. Salerno , G. Albano , et al. 2022. “A Three‐Wave Panel Study on Longitudinal Relations Between Problematic Social Media Use and Psychological Distress During the COVID‐19 Pandemic.” Addictive Behaviors 134: 107430. 10.1016/j.addbeh.2022.107430. PMC9287460 35870439

[jad70237-bib-0007] Donisi, V. , L. Salerno , E. Delvecchio , and A. Brugnera . 2024. “Problematic Social Media Use Among Italian Midadolescents: Protocol and Rationale of the SMART Project.” JMIR Research Protocols 13: e58739. 10.2196/58739. PMC11420604 39248403

[jad70237-bib-0008] van den Eijnden, R. J. J. M. , J. S. Lemmens , and P. M. Valkenburg . 2016. “The Social Media Disorder Scale.” Computers in Human Behavior 61: 478–487. 10.1016/j.chb.2016.03.038.

[jad70237-bib-0009] Essau, C. A. , B. Olaya , X. Anastassiou‐Hadjicharalambous , et al. 2012. “Psychometric Properties of the Strength and Difficulties Questionnaire From Five European Countries.” International Journal of Methods in Psychiatric Research 21, no. 3: 232–245. 10.1002/mpr.1364. PMC6878504 22890628

[jad70237-bib-0010] Favini, A. , F. Culcasi , F. Cirimele , et al. 2024. “Smartphone and Social Network Addiction in Early Adolescents: The Role of Self‐Regulatory Self‐Efficacy in a Pilot School‐Based Intervention.” Journal of Adolescence 96, no. 3: 551–565. 10.1002/jad.12263. 37853581

[jad70237-bib-0011] Gámez‐Guadix, M. 2014. “Depressive Symptoms and Problematic Internet Use Among Adolescents: Analysis of the Longitudinal Relationships From the Cognitive–Behavioral Model.” Cyberpsychology, Behavior and Social Networking 17, no. 11: 714–719. 10.1089/cyber.2014.0226. PMC4238256 25405784

[jad70237-bib-0012] Goodman, R. 1997. “The Strengths and Difficulties Questionnaire: A Research Note.” Journal of Child Psychology and Psychiatry 38, no. 5: 581–586. 10.1111/j.1469-7610.1997.tb01545.x. 9255702

[jad70237-bib-0013] Hamaker, E. L. , R. M. Kuiper , and R. P. P. P. Grasman . 2015. “A Critique of the Cross‐Lagged Panel Model.” Psychological Methods 20, no. 1: 102–116. 10.1037/a0038889. 25822208

[jad70237-bib-0014] Hu, L. , and P. M. Bentler . 1999. “Cutoff Criteria for Fit Indexes in Covariance Structure Analysis: Conventional Criteria Versus New Alternatives.” Structural Equation Modeling: A Multidisciplinary Journal 6, no. 1: 1–55. 10.1080/10705519909540118.

[jad70237-bib-0015] Huang, C. 2022. “A Meta‐Analysis of the Problematic Social Media Use and Mental Health.” International Journal of Social Psychiatry 68, no. 1: 12–33. 10.1177/0020764020978434. 33295241

[jad70237-bib-0016] IBM Corp . 2023. “IBM SPSS Statistics for Windows (Version 29.0.1).” IBM Corp.

[jad70237-bib-0017] Kardefelt‐Winther, D. 2014. “A Conceptual and Methodological Critique of Internet Addiction Research: Towards a Model of Compensatory Internet Use.” Computers in Human Behavior 31: 351–354. 10.1016/j.chb.2013.10.059.

[jad70237-bib-0018] Keles, B. , N. McCrae , and A. Grealish . 2020. “A Systematic Review: The Influence of Social Media on Depression, Anxiety and Psychological Distress in Adolescents.” International Journal of Adolescence and Youth 25, no. 1: 79–93. 10.1080/02673843.2019.1590851.

[jad70237-bib-0019] Kim, J. , R. LaRose , and W. Peng . 2009. “Loneliness as the Cause and the Effect of Problematic Internet Use: The Relationship Between Internet Use and Psychological Well‐Being.” Cyberpsychology & Behavior: The Impact of the Internet, Multimedia and Virtual Reality on Behavior and Society 12, no. 4: 451–455. 10.1089/cpb.2008.0327. 19514821

[jad70237-bib-0020] Kraut, R. , M. Patterson , V. Lundmark , S. Kiesler , T. Mukophadhyay , and W. Scherlis . 1998. “Internet Paradox: A Social Technology That Reduces Social Involvement and Psychological Well‐Being?” American Psychologist 53, no. 9: 1017–1031. 10.1037/0003-066X.53.9.1017. 9841579

[jad70237-bib-0021] Lapierre, M. A. , P. Zhao , and B. E. Custer . 2019. “Short‐Term Longitudinal Relationships Between Smartphone Use/Dependency and Psychological Well‐Being Among Late Adolescents.” Journal of Adolescent Health 65, no. 5: 607–612. 10.1016/j.jadohealth.2019.06.001. 31477510

[jad70237-bib-0022] Lerner, R. M. and Steinberg, L. , eds. 2009. Handbook of Adolescent Psychology, Volume 1: Individual Bases of Adolescent Development. John Wiley & Sons.

[jad70237-bib-0023] Lopes, L. S. , J. P. Valentini , T. H. Monteiro , et al. 2022. “Problematic Social Media Use and Its Relationship With Depression or Anxiety: A Systematic Review.” Cyberpsychology, Behavior and Social Networking 25, no. 11: 691–702. 10.1089/cyber.2021.0300. 36219756

[jad70237-bib-0024] Monacis, L. , V. de Palo , M. D. Griffiths , and M. Sinatra . 2017. “Social Networking Addiction, Attachment Style, and Validation of the Italian Version of the Bergen Social Media Addiction Scale.” Journal of Behavioral Addictions 6, no. 2: 178–186. 10.1556/2006.6.2017.023. PMC5520120 28494648

[jad70237-bib-0025] Muthén, L. K. , and B. O. Muthén . 2024. Mplus 8.11 [Computer Software]. Muthén and Muthén.

[jad70237-bib-0026] Nesi, J. , S. Choukas‐Bradley , and M. J. Prinstein . 2018. “Transformation of Adolescent Peer Relations in the Social Media Context: Part 1—A Theoretical Framework and Application to Dyadic Peer Relationships.” Clinical Child and Family Psychology Review 21, no. 3: 267–294. 10.1007/s10567-018-0261-x. PMC6435354 29627907

[jad70237-bib-0027] Orth, U. , L. L. Meier , J. L. Bühler , et al. 2024. “Effect Size Guidelines for Cross‐Lagged Effects.” Psychological Methods 29, no. 2: 421–433. 10.1037/met0000499. 35737548

[jad70237-bib-0028] Pazdur, M. , D. Tutus , and A. C. Haag . 2025. “Risk Factors for Problematic Social Media Use in Youth: A Systematic Review of Longitudinal Studies.” Adolescent Research Review 10, no. 2: 237–253. 10.1007/s40894-025-00264-4.

[jad70237-bib-0029] Pew Research Center . 2025. “Teens, Social Media and Mental Health.” https://www.pewresearch.org/internet/2025/04/22/teens-social-media-and-mental-health/.

[jad70237-bib-0030] Poli, S. , L. Salerno , E. Delvecchio , et al. 2025. “Problematic Social Media Use in Mid‐Adolescents: A Systematic Review of Intrapersonal and Interpersonal Protective/Risk Factors and Psychosocial Interventions.” Current Addiction Reports 12: 81. 10.1007/s40429-025-00694-z.

[jad70237-bib-0031] Pouwels, J. L. , I. Beyens , L. Keijsers , and P. M. Valkenburg . 2025. “Changing or Stable? The Effects of Adolescents' Social Media Use on Psychosocial Functioning.” Child Development 96, no. 2: 752–770. 10.1111/cdev.14207. PMC11868685 39660973

[jad70237-bib-0032] Raudsepp, L. , and K. Kais . 2019. “Longitudinal Associations Between Problematic Social Media Use and Depressive Symptoms in Adolescent Girls.” Preventive Medicine Reports 15: 100925. 10.1016/j.pmedr.2019.100925. PMC6603436 31304081

[jad70237-bib-0033] Sallquist, J. V. , N. Eisenberg , T. L. Spinrad , et al. 2009. “Positive and Negative Emotionality: Trajectories Across Six Years and Relations With Social Competence.” Emotion (Washington, D.C.) 9, no. 1: 15–28. 10.1037/a0013970. PMC2753671 19186913

[jad70237-bib-0034] Silk, J. S. , L. Steinberg , and A. S. Morris . 2003. “Adolescents' Emotion Regulation in Daily Life: Links to Depressive Symptoms and Problem Behavior.” Child Development 74, no. 6: 1869–1880. 10.1046/j.1467-8624.2003.00643.x. 14669901

[jad70237-bib-0035] Slater, M. D. 2015. “Reinforcing Spirals Model: Conceptualizing the Relationship Between Media Content Exposure and the Development and Maintenance of Attitudes.” Media Psychology 18, no. 3: 370–395. 10.1080/15213269.2014.897236. PMC4565521 26366124

[jad70237-bib-0036] Vannucci, A. , K. M. Flannery , and C. M. Ohannessian . 2017. “Social Media Use and Anxiety in Emerging Adults.” Journal of Affective Disorders 207: 163–166. 10.1016/j.jad.2016.08.040. 27723539

[jad70237-bib-0037] Zhang, J. , J. Ying , Y. Shen , D. Chen , S. Zhan , and J. You . 2025. “Examining the Interrelationships of School Connectedness, Social Anxiety, and Problematic Social Network Use in Adolescents.” Journal of Adolescence 97, no. 3: 687–699. 10.1002/jad.12446. 39572893

[jad70237-bib-0038] Zhou, W. , Z. Yan , Z. Yang , and Z. Hussain . 2023. “Problematic Social Media Use and Mental Health Risks Among First‐Year Chinese Undergraduates: A Three‐Wave Longitudinal Study.” Frontiers in Psychiatry 14: 1237924. 10.3389/fpsyt.2023.1237924. PMC10512716 37743982

